# Amplitude of Low-Frequency Oscillations in First-Episode, Treatment-Naive Patients with Major Depressive Disorder: A Resting-State Functional MRI Study

**DOI:** 10.1371/journal.pone.0048658

**Published:** 2012-10-31

**Authors:** Li Wang, Wenji Dai, Yunai Su, Gang Wang, Yunlong Tan, Zhen Jin, Yawei Zeng, Xin Yu, Wei Chen, Xiaodong Wang, Tianmei Si

**Affiliations:** 1 Institute of Mental Health, Peking University, Beijing, China; 2 The Key Laboratory for Mental Health, Ministry of Health, Beijing, China; 3 Mood Disorders Center, Beijing Anding Hospital, Capital Medical University, Beijing, China; 4 Center for Psychiatric Research, Beijing Huilongguan Hospital, Beijing, China; 5 Department of Radiology, 306 Hospital of People's Liberation Army, Beijing, China; 6 The Key Laboratory of Medical Neurobiology of Chinese Ministry of Health, Hangzhou, China; 7 Max Planck Institute of Psychiatry, Munich, Germany; Bellvitge Biomedical Research Institute-IDIBELL, Spain

## Abstract

**Background:**

Resting-state fMRI is a novel approach to measure spontaneous brain activity in patients with major depressive disorder (MDD). Although most resting-state fMRI studies have focused on the examination of temporal correlations between low-frequency oscillations (LFOs), few studies have explored the amplitude of these LFOs in MDD. In this study, we applied the approaches of amplitude of low-frequency fluctuation (ALFF) and fractional ALFF to examine the amplitude of LFOs in MDD.

**Methodology/Principal Findings:**

A total of 36 subjects, 18 first-episode, treatment-naive patients with MDD matched with 18 healthy controls (HCs) completed the fMRI scans. Compared with HCs, MDD patients showed increased ALFF in the right fusiform gyrus and the right anterior and posterior lobes of the cerebellum but decreased ALFF in the left inferior temporal gyrus, bilateral inferior parietal lobule, and right lingual gyrus. The fALFF in patients was significantly increased in the right precentral gyrus, right inferior temporal gyrus, bilateral fusiform gyrus, and bilateral anterior and posterior lobes of the cerebellum but was decreased in the left dorsolateral prefrontal cortex, bilateral medial orbitofrontal cortex, bilateral middle temporal gyrus, left inferior temporal gyrus, and right inferior parietal lobule. After taking gray matter (GM) volume as a covariate, the results still remained.

**Conclusions/Significance:**

These findings indicate that MDD patients have altered LFO amplitude in a number of regions distributed over the frontal, temporal, parietal, and occipital cortices and the cerebellum. These aberrant regions may be related to the disturbances of multiple emotion- and cognition-related networks observed in MDD and the apparent heterogeneity in depressive symptom domains. Such brain functional alteration of MDD may contribute to further understanding of MDD-related network imbalances demonstrated in previous fMRI studies.

## Introduction

Major depressive disorder (MDD) is highly prevalent and constitutes a pressing public health problem, with a yearly increase in morbidity and a high risk of mortality [1]. Understanding the pathophysiology of MDD is a clear goal in achieving further advances in the therapy of MDD.

In recent years, neuroimaging studies have greatly advanced our understanding of the pathogenesis of MDD. Resting-state fMRI has recently been suggested as a novel neuroimaging tool to investigate MDD because of the pervasive and persistent nature of depressive symptoms. Low-frequency oscillations (LFOs) in the resting-state blood oxygen level dependent (BOLD) signal was thought to be of physiological importance and reflect the spontaneous neural function of the brain [2]. Synchronized LFOs have been successfully used to map the neural networks related to the motor, visual, auditory, language, and default mode systems [3]. Using a series of approaches, such as independent component analysis (ICA), seed-based methods and graph theory analysis, several fMRI studies have been conducted to examine the alterations of network connectivity in MDD patients at rest [4], [5], [6], [7], [8], [9]. Overall, these approaches have led to the proposal that MDD is associated with the dysregulation of various neural networks. These include the cognitive control network (containing regions within the dorsal prefrontal cortex) [9], the affective network (mainly including the amygdala, orbitofrontal cortex, temporal poles, pallidum, and insula) [6], the default mode network (DMN, typically containing the medial prefrontal cortex, inferior temporal gyrus, posterior cingulate, and inferior parietal lobule) [4], [7], and other neural systems associated with risk, reward, attention, and memory processing [5], [6], [7], [8]. Moreover, the connectivities between different neural systems, such as the cognitive control network and the default mode network, are also affected by this illness [5], [9]. These disease-related intraneural and interneural network relations may be associated with the complex emotional and cognitive disturbances in MDD. However, it should be noted that whereas several fMRI studies have focused on the network-level investigation of MDD, few studies have explored BOLD signal changes in the regional spontaneous activity of the brain. Therefore, it is difficult to recognize precisely which regions are altered and which abnormalities of those regions drive the large-scale alterations of network synchronization observed in MDD.

The quantitative measurement of LFO amplitude offers potential tools for the detection of BOLD signal changes in regional spontaneous activity. In the past years, two methods have been developed to examine these amplitudes. One method measures the amplitude of low-frequency ﬂuctuation (ALFF) [10], which is assumed to reflect the absolute intensity of spontaneous brain activity. Although ALFF appears to be a promising method for detecting spontaneous brain activity, certain cisternal areas have also shown significantly higher ALFF, which is likely due to physiological noise. Thus, the fALFF approach was developed to selectively suppress the artifacts from non-specific brain areas and thereby significantly improve the sensitivity and specificity of detecting spontaneous brain activity [11]. Both metrics have been applied to evaluate the LFO amplitude of normal and pathological brains; for example, eyes open versus eyes closed [12], schizophrenia [13], mild cognitive impairment [14], and Alzheimer's disease [14], [15]. To the best of our knowledge, only one fMRI study has investigated the LFO amplitude in adult MDD at rest, and this study focused primarily on the differences between patients with treatment-resistant depression and treatment-responsive depression and only examined the ALFF changes in MDD [16].

While the brain functional changes related to MDD have been well studied, multiple factors such as prolonged exposure to antidepressant medication, illness chronicity, and possible progressive gray matter atrophy may bias the results [17], [18]. Although there are many more fMRI studies that have recruited chronic patients with multiple episodes, studies exploring first-episode and treatment-naive patients with MDD may be more reliable in elucidating the nature of MDD.

The present study sought to compare the intrinsic brain activity of first-episode, treatment-naive, relatively short duration MDD patients with that of matched healthy controls using both the ALFF and fALFF approaches. Given the phenomenon of apparent heterogeneity in depressive symptom domains and the existing findings of neuroimaging studies, we hypothesized that altered LFO amplitude would be found widely across brain regions linked with three types of neural systems in MDD patients: (1) based on the core feature of negative cognitive bias in MDD and the assumed role of hypofunction of the prefrontal cortex in this cognitive bias [19], we predicted that certain regions within the prefrontal cortex, especially in the dorsal and orbital portions, would show reduced activity in MDD patients; (2) based on the involvement of the temporal, parietal, and occipital regions in a series of sensory- and perception-related cognitive functions, such as facial emotional recognition and semantic processing, that have been found to be impaired in MDD [20], [21], [22] and on the abnormal activity of these regions in MDD patients found in previous studies [23], [24], we also expected to find altered activity in these regions; and (3) based on the convergent findings of strengthened responses of limbic regions to negative emotional stimuli in MDD [25], [26], we expected to find increased activity in certain subcortical limbic regions at rest, such as the amygdala.

## Materials and Methods

### Ethics Statement

This research protocol was approved by the Ethics Committee in the Peking University Institute of Mental Health and the Ethics Committee of the Beijing Anding Hospital of Capital Medical University. All of the participants in this study provided written consent.

### Subjects

A total of 36 subjects were recruited from May 2010 to July 2011. Eighteen out- or in-patients with first-episode MDD participated in the current study. All of the patients reported herein were part of a large cohort study of the Chinese population of Han nationality in the Peking University Institute of Mental Health and the Beijing Anding Hospital of Capital Medical University. For patients of the large cohort study, the inclusion criteria included the following: (1) both sexes aged 18–60 years; (2) MDD diagnoses based on the Structured Clinical Interview of the DSM (Diagnostic and Statistical Manual of Mental Disorders)-IV criteria [27] and currently in a first or recurrent episode; and (3) a total HRSD score of no less than 24. The exclusion criteria for patients included the following: (1) other diagnoses in the past year that included organic mental disorders, schizophrenia, schizoaffective disorder, delusional mental disorder, psychotic features coordinated or uncoordinated with mood, bipolar disorder, and substance abuse; (2) a history of major physical illnesses, seizures, brain damage, or any nervous system diseases (including multiple sclerosis, degenerative diseases such as Parkinson's disease and movement disorders); (3) acutely suicidal or homicidal behaviors; and (4) current pregnancy or breastfeeding. A total of 85 patients met the above criteria.

We selected the patients for the current fMRI study from the 85 MDD patients. Additional inclusion criteria included the following: currently in their first episode and duration of depression ≥1 month and ≤24 months. Additional exclusion criteria included the following: currently or previously taking antidepressant or anti-psychotic drugs, currently taking psychotropic medications including benzodiazepines, hypnotics or anticonvulsant agents, a history of electroconvulsive therapy, and contraindications to MRI scan. A total of 18 patients met these additional criteria and completed this fMRI study. On the day of scanning, the severity of depression was assessed using the 17-item Hamilton Depression Rating Scale (HDRS) [28], and the severity of anxiety symptoms was measured using the Hamilton Anxiety Rating Scale (HAMA) [29].

Eighteen age-, gender-, and education-matched control subjects with 17-item HRSD scores less than 7 were recruited from the community. Clinical psychiatrists also assessed the depressive symptoms of the control subjects on the day of scanning. The controls were screened using the non-patient version of the Structured Clinical Interview from the DSM-IV to exclude any history of psychiatric or neurological disorder. Additional exclusion criteria for the control subjects included the following: a history of severe or unstable physical illness, a history of head trauma and loss of consciousness, a family history of major psychiatric or neurological illness in their first-degree relatives, a history of substance abuse or dependence within the last 12 months, acutely suicidal or homicidal tendencies, a history of use of psychotropic medications, current pregnancy or breastfeeding, and contraindications to MRI scanning. All of the subjects were right-handed as determined by the Edinburgh Handedness Test [30], and all of the subjects provided written informed consent before entering the study.

Here, we provide certain background information about the treatment conditions of mental illness in China to aid an understanding of the process of patient recruitment for our study. Although the situation in China has gradually improved, there is still a considerable portion of patients with mental diseases who cannot be identified until their diseases become serious. An earlier survey [1] found that more than 88% of persons with non-psychotic mental disorders, as diagnosed by the DSM-IV criteria, have never received any type of professional help for psychological problems; of the 2,657 persons with mood disorders, 91.7% have never sought help. Moreover, when patients seek treatment for the first time, they frequently prefer to select a large mental health center. When the disease is under control, they are transferred to the community hospitals. Thus, it is easy to understand why we collected all our first-episode, treatment-naive patients in two large mental health centers rather than community hospitals and why certain patients were still unmedicated despite the fact that their diseases have lasted for several months.

### MRI data acquisition and protocol

On the day each patient attended, we completed the patient's recruitment and fMRI scans before they received medication for the first time. All of the images were acquired with a 3.0-T Siemens scanner at the Department of Radiology of the 306th Hospital of the People's Liberation Army. The subjects were instructed to relax, keep their eyes closed, stay awake, remain still, and not think of anything in particular. The subjects' compliance was confirmed after the scanning was completed. First, a localizer scan was acquired for the localization of functional scans. The functional images were recorded axially over 7 min and 6 s using an echo-planar imaging (EPI) sequence with the following parameters: repetition time (TR)/echo time (TE)  = 2000/30 ms, flip angle  = 90°, 30 slices, slice thickness/gap  = 4.0/0.8 mm, voxel size  = 3.3×3.3×4.0 mm3, resolution  = 64×64 matrix, field of view (FOV)  = 210 mm×210 mm, and band-width  = 2232 Hz/pixel. Three-dimensional T1-weighted magnetization-prepared rapid gradient echo (MPRAGE) images were acquired sagittally using the following parameters: TR/TE  = 2300/3.01 ms, resolution  = 256×256 matrix, slice thickness  = 1 mm, 176 slices, FOV  = 256 mm×240 mm, voxel size  = 1×1×1 mm3, flip angle  = 9°, and time  = 6 min and 56 s. In addition to the EPI functional scan and the T1 anatomical scan, a DTI scan was also performed, but the results are not presented in this report. We performed the resting-state EPI scan first to avoid the possible influences of other scans on the BOLD signals. All 36 subjects completed the scans without reporting discomfort during or after the procedure. No subjects fell asleep during scannings. No obvious structural damage was found in any subject based on conventional MRI scans that were examined by two experienced radiologists.

### Functional image preprocessing

The EPI data were preprocessed with the Data Processing Assistant for Resting-State fMRI (DPARSF) [31] that works with SPM5 (http://www.fil.ion.ucl.ac.uk/spm) on the Matlab platform. The first ten volumes of the scanning sessions were removed to allow for scanner calibration and participants' adaptation to the scanning environment. The remaining 200 volumes were analyzed. For each subject, the EPI images were slice-time corrected and realigned. All of the subjects' head movements were less than 1 mm maximum displacement in any direction of x, y and z and less than 1°in any angular dimension. After realignment, all of the data were normalized to Montreal Neurological Institute (MNI) space and resampled with 3×3×3 mm^3^ resolution using transformation parameters that were estimated by a unified segmentation algorithm [32] (see the following “structural image analysis” section for details). Next, smoothing with a Gaussian kernel of 4-mm full-width at half-maximum (FWHM) and removal of linear trends were performed. No band-pass filtering was implemented during preprocessing such that the entire frequency band could be examined in the subsequent analysis.

### ALFF and fALFF analysis

ALFF and fALFF analyses were performed using the DPARSF software as previously described [14], [15]. After the above preprocessing, the fMRI data were temporally band-pass filtered (0.01<f<0.08 Hz) to reduce low-frequency drift and high-frequency respiratory and cardiac noise. The time series of each voxel was transformed into the frequency domain, and the power spectrum was obtained. Because the power of a given frequency is proportional to the square of the amplitude of that frequency component, the square root was calculated at each frequency of the power spectrum, and the averaged square root was then obtained across 0.01–0.08 Hz at each voxel. This averaged square root was taken as the ALFF, which was assumed to reflect the absolute intensity of brain spontaneous activity [10]. Previous studies have found that although the ALFF approach reveals significantly higher ALFF in the posterior cingulate cortex, precuneus, and medial prefrontal cortex, other non-specific areas have also been shown to have significantly higher ALFF, such as the cisterns, the ventricles, and/or the vicinities of large blood vessels [10], [11], which suggests that the original ALFF approach may be sensitive to signal fluctuations from physiological noise irrelevant to brain activity. To overcome these limits of the ALFF approach, a ratio of the power of each frequency at a low-frequency range to that of the entire frequency range (i.e., the fractional ALFF (fALFF)), was computed [11]. Specifically, after the removal of linear trends, the time series for each voxel was transformed into the frequency domain without band-pass filtering. The square root was calculated at each frequency of the power spectrum. The sum of the amplitude across 0.01–0.08 Hz was divided by that of the entire frequency range (0–0.25 Hz in this study). The validity of the fALFF approach in suppressing confounding signals from non-specific areas has been confirmed in a sample of healthy subjects [11].

The ALFF and fALFF computations and further analyses were performed within a gray matter (GM) group mask that was made by setting a threshold of 0.15 on the mean GM map of all 36 subjects. For standardization purposes, each individual ALFF/fALFF map was divided by its own mean ALFF/fALFF values within this mask.

### Structural image analysis

Structural MRI studies have suggested that MDD patients exhibit GM loss in many cerebral regions [33]. The GM loss may produce partial effects on functional images and thus be a potential confounding factor in the assessment of the alteration of brain function in MDD patients. To address this issue, a voxel-based morphometry (VBM) analysis was first performed. In brief, individual T1-weighted anatomical images were coregistered to the mean functional images after head-motion correction using a linear transformation [34]. Next, the transformed structural images were segmented into GM, white matter, and cerebrospinal ﬂuid and spatially normalized in the MNI space using the unified segmentation algorithm [32], which is considered to be capable of solving the circularity problem of registration and tissue classification in optimized VBM. Afterwards, the GM maps were modulated to compensate for the effect of the spatial normalization. The modulated GM maps were then smoothed with a 4-mm Gaussian kernel. The resultant images were used for statistical analysis.

### Statistical analysis

#### ALFF/fALFF analysis without GM correction

To explore the ALFF and fALFF differences between MDD patients and healthy controls, two-sample *t*-tests were performed on the individual normalized ALFF and fALFF maps in a voxel-by-voxel manner. Then, to observe possible clinical relevances, correlation analyses were performed between the ALFF and fALFF maps of MDD patients and the total HRSD scores in a voxel-wise manner.

#### ALFF/fALFF analysis with GM correction

First, regionally specific differences in GM volumes between MDD patients and healthy controls were assessed using two-sample *t*-tests on the smoothed GM maps within the group GM mask. Next, to investigate the effect of GM volume on the functional results, we reanalyzed the relative ALFF/fALFF results (two-sample *t*-tests and correlation analyses), taking GM volume as the covariate in a voxel-wise manner.

For all of the above analyses, significance in the resulting statistical maps was set at 0.05 (corrected for multiple comparisons). The correction standard was determined by Monte Carlo simulations (with the following parameters: individual voxel P value = 0.05, 10,000 simulations, FWHM = 4 mm, with the group GM mask) applied with the Resting-State fMRI Data Analysis Toolkit (REST) of the AlphaSim program [35]. Using this program, a corrected significance level of P<0.05 was obtained for a minimum volume of 1,566 mm^3^. This process enabled the identification of significant differences in the LFO amplitude between MDD patients and healthy controls. Our study adopted a 4-mm smoothing kernel in data preprocessing due to the fact that there are no definite approaches to estimate the most effective FWHM, though the use of a 4-mm smoothing kernel might overestimate the cluster significance of between-group differences. Additionally, although age was not significantly different between the two groups, we still took age as a covariate to avoid an undetected impact on imaging results. All of the statistical analyses were performed using the REST software.

## Results

### Demographic and Clinical Comparisons

The demographic and clinical data are presented in Table 1. There were no significant differences between MDD patients and healthy controls in gender, age, years of education, or handedness. An examination of movement parameters found no significant differences in maximum movement values in any plane of translation (x, y, or z) (*P*>0.05) or any plane of rotation (roll, pitch, or yaw) (*P*>0.05) between the two groups.

### ALFF/fALFF without GM correction

#### ALFF

**Figure 1:**
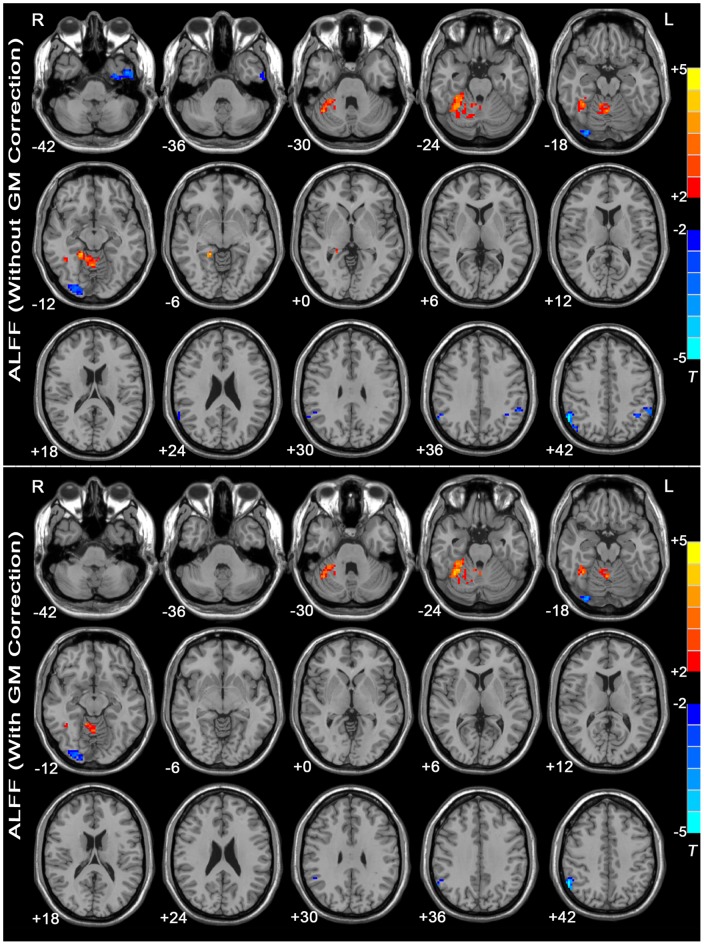
T-statistic maps of ALFF between MDD patients and healthy controls. The threshold was set at *P*<0.05 (corrected). *T*-score bars are shown at right. Warm colors indicate increased ALFF in the MDD patients compared with the controls, and cold colors indicate the opposite. The numbers at the bottom left of the images refer to the z-coordinates in the standard space of the MNI template. The upper map depicts the results without GM correction. The lower map shows the results with GM correction. Details are provided in Table S1.

Figure 1 shows the ALFF differences between the MDD patients and the healthy controls. Compared with the healthy controls, the MDD patients showed significant ALFF increases in the right fusiform gyrus and the right anterior and posterior lobes of the cerebellum. The regions showing decreased ALFF in MDD patients included the left inferior temporal gyrus, bilateral inferior parietal lobule, and right lingual gyrus. See Table S1 for a list of these regions.

**Table 1:** Demographics and clinical characteristics of the subjects.

Variables	MDD (*n* = 18)	HCs (*n* = 18)	*P* value
Gender (male/female)	9/9	9/9	>0.99^a^
Age (years)	34±13	35±12	0.784^b^
Education (years)	13±2	14±2	0.527^b^
Handedness (right/left)	18/0	18/0	>0.99^a^
Illness duration (months)	5±4	N/A	
Total HRSD score	25±5	4±3	
Total HAMA score	17±6	N/A	

Abbreviations: MDD, Major Depressive Disorder. HCs, Healthy Controls.

HRSD: Hamilton Rating Scale for Depression.

HAMA: Hamilton Anxiety Rating Scale.

N/A: not applicable.

a and ^b^ indicate the *P* values for the chi-squared test and two-sample *t*-test, respectively.

#### fALFF

**Figure 2:**
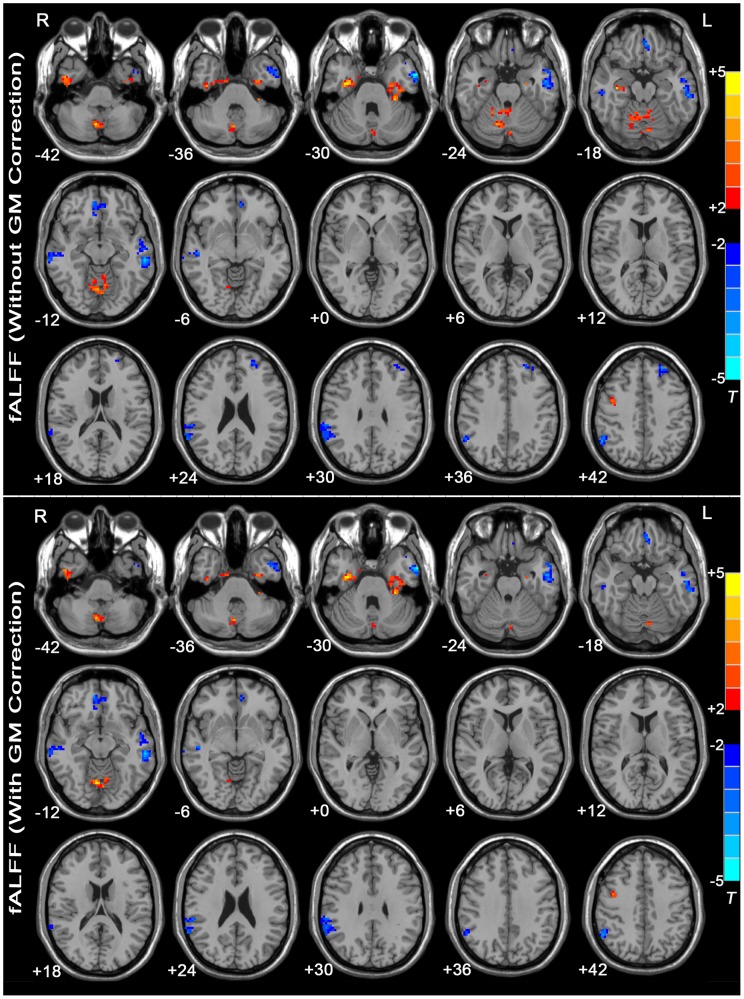
T-statistic maps of fALFF between MDD patients and healthy controls. The threshold was set at *P*<0.05 (corrected). *T*-score bars are shown at right. Warm colors indicate increased fALFF in the MDD patients compared with the controls, and cold colors indicate the opposite. The numbers at the bottom left of the images refer to the z-coordinates in the standard space of the MNI template. The upper map depicts the results without GM correction. The lower map shows the results with GM correction. Details are provided in Table S2.

Figure 2 shows the fALFF differences between the MDD patients and the healthy controls. Compared with the healthy controls, the MDD patients showed significant fALFF increases in the right precentral gyrus, right inferior temporal gyrus, bilateral fusiform gyrus, and bilateral anterior and posterior lobes of the cerebellum. The regions showing decreased fALFF in the MDD patients included the left dorsolateral prefrontal cortex, bilateral medial orbitofrontal cortex, bilateral middle temporal gyrus, left inferior temporal gyrus, and right inferior parietal lobule. See Table S2 for a list of these regions.

### VBM

Compared with the controls, the MDD patients showed a significant GM volume reduction in one frontal region localized in the left inferior frontal gyrus, several lateral temporal regions, the right fusiform gyrus, and the right insula. For details, see Figure S1 and Table S3.

### ALFF/fALFF with GM correction

As shown in Figs 1 and 2, using the GM volume as a covariate in the ALFF/fALFF analyses produced results similar to the analyses without GM correction, but the statistical significance of the group differences was partially reduced, and certain regions failed to survive the correction for multiple comparisons. For details, see Tables S1 and S2.

### Correlations between ALFF/fALFF and illness severity

No significant correlations were found between the ALFF/fALFF values of any region and the total HRSD scores before and after the GM correction.

## Discussion

Using both the ALFF and fALFF measures, the current study examined resting-state cerebral function in a cohort of first-episode, treatment-naive patients with MDD. We found disrupted LFO amplitude (i.e., ALFF and fALFF) in multiple brain regions in MDD patients, with decreased amplitude distributed over the frontal, temporal, parietal, and occipital regions. In contrast with the previous speculation, we did not observe enhanced activity in any region of the limbic system but found increased activity primarily in the cerebellum. Thus, in contrast to the functional connectivity methods that measure the temporal synchronization between brain regions, the ALFF and fALFF approaches help to localize the brain functional alterations in MDD and provide additional data in understanding of the disturbances of MDD-related neural networks demonstrated in previous fMRI studies [4], [5], [6], [7], [8], [9].

### Frontal region

The frontal lobe is one of the regions that is most consistently identified as associated with MDD [36], [37]. According to Beck's cognitive model [38], MDD patients display a cognitive bias towards negative information and away from positive information, which contributes to the maintenance of a depressed mood. Distinct subregions of the frontal cortex play critical roles in the cognitive bias of MDD. The dorsal lateral prefrontal cortex (DLPFC) is a crucial member of the cognitive control network that modulates the effortful control of negative emotion [39], whereas the ability to experience positive affect has been linked to the brain systems that mediate reward or motivation, including the orbitofrontal cortex (OFC) [40]. Using specific task stimuli, disturbances of brain systems related to cognitive control and reward function have typically been found in MDD, and the DLPFC and OFC have been shown to be important nodes mediating the dysfunction of these systems [38], [41]. Moreover, resting-state studies using PET have consistently revealed reduced activity in both the DLPFC and OFC of MDD patients [42], [43]. Although similar to the findings of previous PET studies, our study demonstrated the functional deficits of two frontal regions in MDD with novel functional parameters using functional MRI. We further speculate that the hypoactivity of the left DLPFC and bilateral OFC observed in our patients may underlie the disturbances of the cognitive control and reward brain systems, which would contribute to the persistent negative feelings experienced by MDD patients. Of note, these frontal abnormalities were not detected with the ALFF measure, which may indicate that fALFF, as the normalized ALFF, can provide a more specific measurement of the LFO phenomena.

### Temporal, Parietal, and Occipital regions

Although there has been considerable focus on the frontal cortex in MDD, the current study identified the changes in a number of lateral temporal, parietal, and occipital regions, as well as the pre-central gyrus as outlined below. These regions are primarily related to sensory-motor processing but also participate in a series of higher cognitive functions. The altered LFO amplitude in these regions detected in our patients may be potential neural mechanisms mediating the imbalance of these regions' related functional networks in MDD.

### Temporal region

In addition to primary auditory function [44], the lateral temporal regions also play important roles in social cognition and emotional processing [45], [46]. In response to positive social stimuli, MDD patients showed reduced activity in structures including the prefrontal, temporal, and parietal cortices, the insula, and the basal ganglia [45]. Moreover, abnormal modulation of frontotemporal effective connectivity has been observed in patients with remitted MDD when stimulated by happy and sad facial expressions [46]. Evidence from R-fMRI studies also supports the functional abnormality of the lateral temporal regions in MDD, as increased regional homogeneity (Reho) has been found in the left superior temporal gyrus, right middle temporal gyrus, and right inferior temporal gyrus of MDD patients [47], [48]. In contrast to the Reho approach, which measures the degree of regional coherence of LFOs, our study revealed the abnormality of lateral temporal regions in MDD in terms of LFO amplitude. Taken together, the present and previous findings suggest that the abnormalities of the temporal regions, especially the lateral portions, may represent part of the disturbed neural networks associated with the aberrant social functioning and negative emotional processing observed in MDD.

### Parietal region

The inferior parietal lobule (IPL) integrates information from different sensory modalities and is a region that has been repeatedly identified to be within the posterior portion of the default mode network, which is assumed to be associated with self-related cognitive processing, such as autobiographical memory [49], [50], [51]. A previous R-fMRI study proposed that the deficits in episodic memory retrieval in MDD might lead to reduced functional connectivity in the posterior regions of the DMN [5]. More recently, another R-fMRI study supported that speculation with findings of decreased functional connectivity within the posterior regions of the DMN, including the IPL, which is significantly correlated with autobiographical memory performance in MDD patients [52]. The present study found that MDD patients have reduced ALFF in the bilateral IPL and reduced fALFF in the right IPL. Together with the above-mentioned findings, we speculate that the hypoactivity of the IPL may contribute to the disconnection of the posterior DMN, thereby leading to the impairment of autobiographical memory, an MDD-related pathopsychological characteristic related to the self.

We additionally found a fALFF increase in the right pre-central gyrus (PG) of MDD patients. We discussed this region together with the IPL because we assume that the pathway between the PG and IPL is a part of the functional systems involved in aspects of semantic processing, which can be impaired in MDD patients [21]. Given that a previous fMRI study showed reduced functional connectivity between the PG and IPL in MDD patients at rest [5], it is possible that the increased activity of the right PG and reduced activity of the IPL observed in our patients may reflect an uncoupling of the two regions, which may contribute to the impaired semantic processing found in MDD.

### Occipital region

In the occipital lobe, we found increased ALFF in the right fusiform gyrus, decreased ALFF in the right lingual gyrus, and increased fALFF in the bilateral fusiform gyrus in MDD patients. The fusiform gyrus and lingual gyrus are within the visual recognition network, and both regions are thought to be involved in the perception of emotions during the presentation of facial stimuli [5], [53]. A previous R-fMRI study demonstrated an uncoupling of the visual recognition network between the right lingual gyrus and right fusiform gyrus in MDD [5]. Moreover, decreased nodal centralities [7] and white matter integrity [54] in occipital regions have been observed in first-episode, drug-naive MDD patients. Therefore, it can be postulated that the altered LFO amplitude of the occipital regions observed in our patients may underlie the disturbances of the visual recognition network in MDD and may be a neural basis for the impaired facial emotion processing of MDD. Indeed, altered responsiveness to facial emotional stimuli has been used as one of the biomarkers for the early diagnosis of MDD [55].

### Cerebellum

The study also produced an interesting, if tentative, finding regarding the role of the cerebellum. For a long time, the cerebellum was not considered in neurobiological studies. However, recent findings suggest that the cerebellum appears to be more important than previously thought. In addition to its motor function [56], the cerebellum has anatomical connections with multiple areas of the frontal cortex and limbic regions, which are critical for its involvement in emotional and cognitive processing [57]. Studies focusing on functional integration in MDD have demonstrated decreased connectivity between the cerebellum and the OFC in adult MDD patients during the recognition of negative expressions [58] and a fronto-cerebellar dysregulation in adolescents with MDD that is associated with both poor performance in motivated attention and depressive symptoms [59]. We found substantially increased ALFF and fALFF in the cerebellum in MDD patients at rest. The simultaneous increased activity in the cerebellum and reduced activity in the frontal regions observed in our patients provide strong support for the imbalance of fronto-cerebellar loops previously observed in MDD [58], [59]. The dysfunction of the cerebellum, from both regional and network perspectives, may partially mediate the emotional and cognitive symptoms of MDD. Further investigations are required to evaluate the role of the cerebellum in the pathophysiology of MDD.

Unexpectedly, we did not find increased activity in any limbic regions in the MDD patients. Many task-state fMRI studies have found that compared with normal subjects, MDD patients had stronger reactions to negative stimuli in subcortical limbic regions, most commonly in the amygdala, thalamus, and hippocampus [25], [26], [60]. However, it seems that significantly fewer findings of abnormality in these regions have been reported in R-fMRI studies. Several R-fMRI studies investigating regional brain function in MDD did not find activity changes in the amygdala, thalamus, or hippocampus [48], [61], [62], [63]. This result may indicate that the functional alterations of the limbic regions are more prone to being triggered by external emotional stimuli. Task-related fMRI studies need to be performed in the future to clarify this speculation.

No significant correlations were found between ALFF/fALFF measurements in any region and illness severity. This phenomenon intuitively suggests that neither the ALFF nor the fALFF can be used as a quantitative marker for the assessment of depressive severity at this stage, although these indicators are helpful for the localization of functionally aberrant regions. However, the failure to find any correlation may be due to the relatively small sample size and narrow HRSD score range in this study.

Furthermore, to examine the possible effect of brain structural atrophy or variance on functional results, we reanalyzed the functional alterations in MDD using GM volume as a covariate. After taking GM volume as the covariate, we found that the patterns of ALFF and fALFF alteration were similar to those found without the GM correction, although the results were less significant. This observation suggests that the results of ALFF and fALFF can reﬂect the alterations of spontaneous brain activity in MDD patients.

### Limitations and Further Considerations

The major strength of this study is the recruitment of first-episode, treatment-free MDD patients. However, several issues need to be addressed. First, the sample size is relatively small, which limits the statistical power; thus, the findings of our study should be considered to be preliminary. Additionally, due to the cross-sectional design, whether these abnormal LFO amplitudes change dynamically after therapy needs to be explored in longitudinal studies. Second, similarly to other R-fMRI studies, we could not completely eliminate the effects of physiological noise, such as cardiac and respiratory fluctuations, at the relatively low sampling rate used (TR = 2 s). Future studies should simultaneously record the cardiac and respiratory rates to deal with these potential confounding variables. Third, we cannot exclude the possibility that the group differences in LFO amplitude resulted from different spontaneous thoughts and cognitive processing between groups during scanning. In future studies, the development of scales that can effectively examine spontaneous thoughts during scans will be helpful for examining the behavioral group differences in resting-state studies. A further limitation of this study is the lack of correlation between the brain regional changes with neuropsychological measures eg cognitive function, sensory-motor function, working and autobiographical memory, and affective processing. Finally, we only examined the alteration of regional brain function in MDD patients, but a combined analysis of multimodal imaging data will produce more fruitful information toward an understanding of the pathological mechanism of MDD.

In summary, using two new functional parameters, our study identified a series of brain regions closely associated with MDD. These aberrant regions may be partially interrelated with the disturbances of multiple emotion- and cognition-related functional networks observed in MDD. These large-scale functional alterations in brain regions are in keeping with the phenomenon of apparent heterogeneity of depressive symptom domains. Therefore, the current findings complement previous studies investigating network integration in MDD and contribute to a comprehensive understanding of the pathophysiology of MDD.

## Supporting Information

Figure S1 **T-statistic map of GM volume between MDD patients and healthy controls.** The threshold was set at *P*<0.05 (corrected). *T*-score bars are shown at right. Cold colors indicate that MDD patients had reduced GM volume compared with the controls. The numbers at the bottom left of the images refer to the z-coordinates in the standard space of the MNI template. Details are provided in Table S3.
(TIF)

Table S1 **Regions showing ALFF differences between MDD patients and healthy controls.**
(DOC)

Table S2 **Regions showing fALFF differences between MDD patients and healthy controls.**
(DOC)

Table S3 **Regions showing differences in GM volume between MDD patients and healthy controls.**
(DOC)
