# Supplementary figures and images for: Amplitude of Low-Frequency Oscillations in First-Episode, Treatment-Naive Patients with Major Depressive Disorder: A Resting-State Functional MRI Study
Source: PLoS One. 2012 Oct 31;7(10):e48658. doi: 10.1371/journal.pone.0048658 (PMC3485382; doi:10.1371/journal.pone.0048658)

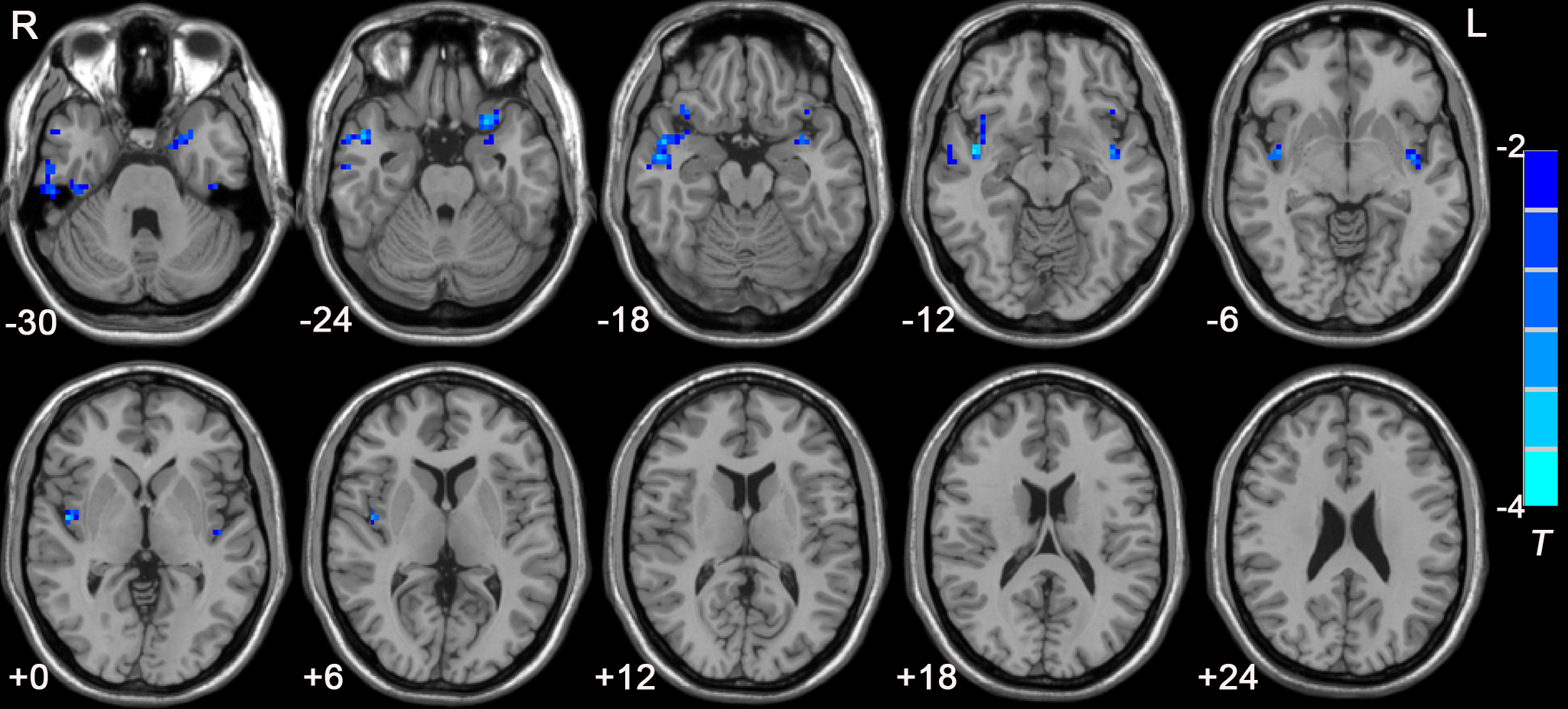

Supplement: Figure S1 — T-statistic map of GM volume between MDD patients and healthy controls. The threshold was set at P<0.05 (corrected). T-score bars are shown at right. Cold colors indicate that MDD patients had reduced GM volume compared with the controls. The numbers at the bottom left of the images refer to the z-coordinates in the standard space of the MNI template. Details are provided in Table S3. (TIF) [file pone.0048658.s001.tif]
