# Supplementary material for: Amplitude of Low-Frequency Oscillations in First-Episode, Treatment-Naive Patients with Major Depressive Disorder: A Resting-State Functional MRI Study
Source: PLoS One. 2012 Oct 31;7(10):e48658. doi: 10.1371/journal.pone.0048658 (PMC3485382; doi:10.1371/journal.pone.0048658)
Supplement: Table S1 — Regions showing ALFF differences between MDD patients and healthy controls. (DOC) [file pone.0048658.s002.doc]

**Table S1. Regions showing ALFF differences between MDD patients and healthy controls.**

| **Regions** | **BA** | **Cluster size (mm^3^)** | ***T* scores of peak voxel** | **Coordinates of peak voxel in MNI space (x, y, z)** |
| --- | --- | --- | --- | --- |
| Without GM Correction |  |  |  |  |
| L ITG | 20 | 2214 | -3.01 | -30 -8 -42 |
| L IPL | 40 | 2322 | -3.94 | -57 -45 45 |
| R IPL | 40 | 2349 | -4.39 | 57 -51 48 |
| R LG | 18 | 2160 | -3.54 | 33 -90 -18 |
| R FG | 37 | 2187 | 3.02 | 36 -48 -18 |
| R ALC/PLC | N/A | 6588 | 3.63 | 33 -40 -28 |
| With GM Correction |  |  |  |  |
| L ITG^a^ | 20 | 1377 | -3.19 | -51 -3 -42 |
| L IPL^a^ | 40 | 1053 | -3.93 | -57 -45 45 |
| R IPL | 40 | 1863 | -4.38 | 57 -51 48 |
| R LG | 18 | 1917 | -3.61 | 30 -90 -15 |
| R FG | 37 | 1620 | 2.86 | 39 -48 -16 |
| R ALC/PLC | N/A | 3969 | 3.60 | 33 -39 -27 |

Abbreviations: L: left. R: right. ITG: inferior temporal gyrus. IPL: inferior parietal lobule. LG: [lingual gyrus](http://en.wikipedia.org/wiki/Lingual_gyrus). FG: fusiform gyrus, ALC: anterior lobe of cerebellum. PLC: posterior lobe of cerebellum. BA: Brodmann's area. N/A: not applicable. *T*: statistical value of peak voxel showing ALFF differences between the two groups (negative values: MDD<HCs; positive values: MDD>HCs). MNI: Montreal Neurological Institute Coordinate System or Template; x, y, z: coordinates of primary peak locations in the MNI space. ^a^ The regions survived the height but not the extent threshold.
