# Supplementary material for: Amplitude of Low-Frequency Oscillations in First-Episode, Treatment-Naive Patients with Major Depressive Disorder: A Resting-State Functional MRI Study
Source: PLoS One. 2012 Oct 31;7(10):e48658. doi: 10.1371/journal.pone.0048658 (PMC3485382; doi:10.1371/journal.pone.0048658)
Supplement: Table S2 — Regions showing fALFF differences between MDD patients and healthy controls. (DOC) [file pone.0048658.s003.doc]

**Table S2. Regions showing fALFF differences between MDD patients and healthy controls.**

| **Regions** | **BA** | **Cluster size (mm^3^)** | ***T* scores of peak voxel** | **Coordinates of peak voxel in MNI space(x, y, z)** |
| --- | --- | --- | --- | --- |
| Without GM Correction |  |  |  |  |
| L DLPFC | 9 | 1593 | -3.61 | -24 45 42 |
| mOFG | 11 | 1944 | -3.52 | -3 42 -18 |
| R PG | 6 | 2565 | 3.86 | 45 0 51 |
| L MTG/ITG | 21/20 | 6318 | -4.94 | -60 0 -30 |
| R MTG | 21 | 2025 | -3.31 | 69 -27 -9 |
| R IPL | 40 | 3969 | -3.65 | 57 -54 39 |
| L FG | 37 | 1620 | 2.75 | -28 -14 -30 |
| R FG/ITG | 37/20 | 3213 | 3.95 | 33 -12 -30 |
| ALC | N/A | 5211 | 3.59 | -30 -33 -30 |
| PLC | N/A | 6048 | 3.24 | 13 -66 -24 |
| With GM Correction |  |  |  |  |
| L DLPFC^a^ | 9 | 1485 | -3.64 | -24 45 39 |
| mOFG | 11 | 1917 | -3.57 | 9 48 -12 |
| R PG | 6 | 2322 | 4.10 | 30 -15 66 |
| L MTG/ITG | 21/20 | 5589 | -4.83 | -60 -30 -15 |
| R MTG | 21 | 1782 | -3.27 | 69 -26 -9 |
| R IPL | 40 | 3780 | -3.77 | 57 -54 39 |
| L FG | 37 | 1728 | 2.71 | -27 -16 -30 |
| R FG/ITG | 37/20 | 2430 | 3.91 | 36 -12 -30 |
| PLC | N/A | 3672 | 3.96 | -9 -63 -54 |

Abbreviations: L: left. R: right. DLPFC: [dorsolateral prefrontal cortex](http://en.wikipedia.org/wiki/Dorsolateral_prefrontal_cortex). mOFC: medial [orbitofrontal](http://en.wikipedia.org/wiki/Orbitofrontal_cortex) cortex. PG: precentral gyrus. MTG: middle temporal gyrus. ITG: inferior temporal gyrus. IPL: inferior parietal lobule. FG: fusiform gyrus. ALC: anterior lobe of cerebellum. PLC: posterior lobe of cerebellum. BA: Brodmann's area. N/A: not applicable. *T*: statistical value of peak voxel showing fALFF differences between the two groups (negative values: MDD<HCs; positive values: MDD>HCs). MNI: Montreal Neurological Institute Coordinate System or Template; x, y, z: coordinates of primary peak locations in the MNI space. ^a^ The regions survived the height but not the extent threshold.
