# Supplementary material for: Amplitude of Low-Frequency Oscillations in First-Episode, Treatment-Naive Patients with Major Depressive Disorder: A Resting-State Functional MRI Study
Source: PLoS One. 2012 Oct 31;7(10):e48658. doi: 10.1371/journal.pone.0048658 (PMC3485382; doi:10.1371/journal.pone.0048658)
Supplement: Table S3 — Regions showing differences in GM volume between MDD patients and healthy controls. (DOC) [file pone.0048658.s004.doc]

**Table S3. Regions showing differences of GM volume between MDD patients and healthy controls.**

| **Regions** | **BA** | **Cluster size (mm^3^)** | ***T* scores of peak voxel** | **Coordinates of peak voxel in MNI space(x, y, z)** |
| --- | --- | --- | --- | --- |
| R ITG/FG | 20/37 | 1809 | -3.21 | 45 -9 -39 |
| L STG/IFG | 22/47 | 2484 | -3.48 | -39 -6 -9 |
| R MTG/STG/IC | 21/22/13 | 2322 | -3.63 | 51 6 -21 |

Abbreviations: L: left. R: right. ITG: inferior temporal gyrus. FG: fusiform gyrus. STG: superior temporal gyrus. IFG: [inferior frontal gyrus](http://en.wikipedia.org/wiki/Inferior_frontal_gyrus). MTG: middle temporal gyrus. IC: [insular cortex](http://en.wikipedia.org/wiki/Insular_cortex). BA: Brodmann's area. *T*: statistical value of peak voxel showing differences of GM volume between the two groups (negative values: MDD<HCs). MNI: Montreal Neurological Institute Coordinate System or Template; x, y, z: coordinates of primary peak locations in the MNI space.
